# Interaction Between Implant Macrogeometry and Osteotomy Preparation on Immediate Mechanical Fixation and Regional Thread Alterations: An Ex Vivo Biomechanical Study

**DOI:** 10.3390/bioengineering13101108

**Published:** 2026-09-23

**Authors:** Almudena Banegas-Cano, Arturo Sánchez-Pérez, Alfonso Jornet-García, José María Montoya-Carralero, María José Moya-Villaescusa

**Affiliations:** Periodontology Unit, Faculty of Medicine, University of Murcia, 30008 Murcia, Spain; almudena.banegas@gmail.com (A.B.-C.); alfjornet@gmail.com (A.J.-G.); icuect@hotmail.com (J.M.M.-C.); mjm.villaescusa@um.es (M.J.M.-V.)

**Keywords:** dental implants, implant macrogeometry, osteotomy preparation, reverse torque, biomechanical fixation, implant threads, osseodensification, ex vivo model

## Abstract

Immediate implant mechanics may depend on the interaction between implant macrogeometry and osteotomy preparation, but their combined influence on complementary measures of immediate fixation and regional thread alterations remains insufficiently characterized. This ex vivo study evaluated two implant macrogeometries and four osteotomy techniques in 80 bovine rib specimens (*n* = 10 per group). Reverse torque resistance remained the primary mechanical outcome; prospectively recorded insertion torque and resonance frequency analysis (implant stability quotient [ISQ]) were incorporated in the revision as complementary immediate mechanical outcomes. Thread alterations were assessed qualitatively after removal in the cervical, body, and apical regions. Insertion torque was significantly influenced by macrogeometry and preparation technique, without a significant interaction. Reverse torque resistance showed significant effects of macrogeometry, preparation technique, and their interaction (all *p* < 0.001). ISQ showed a significant macrogeometry-by-technique interaction. Insertion torque correlated strongly with reverse torque resistance (r = 0.645, *p* < 0.001), whereas ISQ showed a more moderate association (r = 0.370, *p* < 0.001), supporting related but non-interchangeable mechanical constructs. Cervical alterations were mainly associated with undersized drilling, whereas apical alterations occurred predominantly in the Quattro design; none were observed in the implant body. External surface temperature showed no overall change or consistent technique-dependent increase. Under these experimental conditions, implant macrogeometry and osteotomy preparation acted as interdependent determinants of immediate mechanical behavior and were associated with distinct regional patterns of post-removal, visually detectable thread alterations.

## 1. Introduction

The immediate mechanical fixation of a dental implant results from the interaction among the implant, the recipient bone, and the surgical procedure used to prepare the osteotomy. This initial mechanical condition is particularly relevant when the trabecular structure provides limited resistance to implant insertion and loading. Primary implant stability is commonly characterized using insertion torque or resonance frequency analysis; however, these measurements reflect different aspects of the implant–bone system, are only moderately correlated, and should not be considered interchangeable or independently predictive of clinical outcome [1,2,3,4,5].

Several modifications of conventional drilling have been proposed to increase mechanical engagement under conditions of limited bone density. Undersized drilling reduces the diameter of the final osteotomy relative to the implant, thereby increasing mechanical interference during placement, whereas osteotome-mediated expansion progressively displaces and compacts the surrounding bone [6,7,8]. Osseodensification uses specially designed burs to preserve and laterally compact trabecular bone rather than removing it primarily through conventional cutting [9,10,11,12,13,14,15,16]. Experimental, clinical, and evidence-synthesis studies indicate that these approaches can increase insertion torque, implant stability quotient values, or early mechanical fixation under selected conditions, particularly in low-density bone [6,8,9,10,11,12,13,14,15,16,17]. Nevertheless, the magnitude and persistence of these effects are heterogeneous and depend on the bone model, osteotomy dimensions, comparator protocol, and implant design; current evidence does not support a universally superior preparation technique [2,7,8,14,15,16].

Implant macrogeometry is another major determinant of immediate mechanical behavior. Implant shape, diameter, thread depth, pitch, flank configuration, cutting features, and cervical microthreads influence bone–implant contact and the biomechanical behavior of the implant–bone system [2,18,19,20,21,22]. Designs with deeper or more pronounced threads may improve mechanical engagement in trabecular bone, although the resulting mechanical response depends on both the implant geometry and the characteristics of the prepared site [19,20,21].

Consequently, implant macrogeometry and osteotomy preparation should not be regarded as independent variables. The same preparation protocol may produce different mechanical responses when combined with different implant designs.

Most investigations evaluating modified osteotomy techniques have focused on insertion torque, resonance frequency analysis, removal torque, or histomorphometric outcomes [6,8,9,10,17,20,22]. Less attention has been devoted to changes in implant thread morphology occurring after placement and removal. Experimental evidence indicates that implant macrogeometry and bone density can influence surface topography changes and titanium release during insertion [23]. High torque values have also been associated with mechanical alterations in implant connection components, although these findings concern different implant regions and cannot be directly extrapolated to external thread morphology [24].

Regional assessments of visually detectable thread alterations may provide exploratory information regarding how implant designs respond to the mechanical environment created by different preparation methods. However, such observations cannot be interpreted as evidence of functional damage, impaired osseointegration, fatigue failure, or reduced clinical survival unless those outcomes are specifically evaluated.

Reverse torque resistance provides an additional experimental measure of immediate mechanical engagement. Unlike insertion torque, which records the rotational force required to advance an implant, reverse torque reflects the resistance encountered when rotation is initiated in the opposite direction. Experimental studies have used insertion and removal torque measurements to compare the mechanical consequences of different osteotomy protocols and implant macrogeometries in standardized or low-density models [20,22,25]. Nevertheless, reverse torque is not interchangeable with insertion torque, resonance frequency analysis, or micromotion, nor is it a validated clinical predictor of osseointegration, survival, or success [4,5].

Accordingly, the aim of this ex vivo biomechanical study was to evaluate the individual and interactive effects of implant macrogeometry and the osteotomy preparation technique on immediate reverse torque resistance and on the regional distribution of visually detectable implant thread alterations after placement and removal. It was hypothesized that the mechanical effect of osteotomy preparation and the resulting pattern of regional thread alterations would differ according to implant macrogeometry.

## 2. Materials and Methods

### 2.1. Study Design

The experimental factors included implant macrogeometry, with two levels, and the osteotomy preparation technique, with four levels. The combination of these factors generated eight experimental groups. A total of 80 implants were evaluated, with 10 independent implant–bone specimens in each group.

Each bovine rib sample received only one implant. Therefore, each implant–rib combination constituted a separate experimental unit, and all observations were considered statistically independent.

Reverse torque resistance measured immediately after implant placement remained the primary mechanical outcome. In response to peer review, prospectively recorded insertion torque and resonance frequency analysis expressed as implant stability quotient (ISQ), available for all 80 experimental units in the original dataset, were incorporated as complementary immediate mechanical outcomes. Periotest values (Siemens AG, Bensheim, Germany), also available in the original dataset, were reanalyzed as a secondary complementary mechanical measure. The presence or absence of visually detectable thread alterations in the cervical, body, and apical regions was analyzed as an exploratory structural outcome. External temperature measurements obtained during osteotomy preparation and implant insertion were considered a secondary procedural outcome.

All osteotomies, implant placements, reverse torque measurements, and implant removals were performed by the same experienced implant surgeon to minimize operator-dependent variability.

### 2.2. Preliminary Selection of the Experimental Bone Model

Before a definitive experiment was conducted, several animal-derived bone substrates were preliminarily assessed to identify a model with drilling and handling characteristics resembling those encountered clinically in low-density posterior maxillary bone.

The substrates tested during this preliminary phase included bovine vertebrae, rabbit tibiae, porcine ribs, and bovine ribs. All the candidate substrates were evaluated by the same experienced surgeon, who subsequently performed the definitive experimental procedures.

The preliminary assessment was based on tactile perception during drilling, osteotomy preparation, and implant insertion. Among the substrates tested, bovine rib bone was considered by the operator to provide the closest tactile approximation to the resistance clinically perceived in low-density posterior maxillary bone. Bovine ribs were therefore selected for the definitive experiment.

This preliminary selection was based on the subjective tactile assessment of a single experienced operator and was not supported by computed tomography, histomorphometry, mechanical testing, or calibrated measurements of bone density. Consequently, the definitive model is described as an ex vivo bovine rib model with handling characteristics perceived to resemble low-density maxillary bone. It was not objectively classified as human type III or type IV bone.

### 2.3. Bone Specimens

Eighty fresh bovine rib specimens obtained from a local slaughterhouse were used in the experiments. The specimens originated from animals slaughtered for food production and were considered animal-derived byproducts of the food industry. No animals were sacrificed specifically for this investigation, and no experimental intervention was performed in living animals.

Ethical review and approval were not required because the bovine rib specimens were food-industry byproducts obtained after slaughter for human consumption, no animals were sacrificed specifically for this investigation, and no experimental intervention was performed in living animals. Accordingly, the study did not involve live-animal experimentation requiring animal ethics committee approval.

The specimens were visually inspected before use, and sites showing evident cortical damage, structural defects, or insufficient dimensions for implant placement were excluded.

Before osteotomy preparation and implant insertion, the rib specimens were maintained in a temperature-controlled water bath at 30 ± 1 °C. This procedure was intended to reduce differences in baseline specimen temperature and to maintain comparable experimental conditions throughout the study.

One osteotomy was prepared in each rib, and only one implant was inserted into each sample. The 80 ribs therefore provided 80 anatomically and statistically independent experimental units.

### 2.4. Dental Implants

Two commercially available implant macrogeometries from the same manufacturer were evaluated: V2 and Quattro implants (Ticare^®^, Mozo Grau, Valladolid, Spain).

All the implants had identical nominal dimensions of 3.75 mm in diameter and 13 mm in length. They were manufactured from commercially pure grade IV titanium and underwent the same resorbable blasting media surface treatment. Maintaining the implant dimensions, material, manufacturer, and surface treatment constant allowed macrogeometry to be evaluated as the principal implant-related experimental factor.

The V2 design presented a conventional thread configuration, with a greater number of cervical microthreads and a less pronounced thread profile along the implant body. The Quattro design incorporated fewer cervical microthreads and a deeper and more aggressive thread configuration, particularly in the body and apical regions.

The two implant designs are illustrated in Figure 1.

### 2.5. Experimental Groups

The experimental design crossed two implant macrogeometries (V2 and Quattro) with four osteotomy preparation techniques (conventional drilling, undersized drilling, bone expansion using osteotomes, and osseodensification using densifying burs), generating eight experimental groups.

The eight groups were V2-conventional, V2-undersized, V2-expansion, V2-osseodensification, Quattro-conventional, Quattro-undersized, Quattro-expansion, and Quattro-osseodensification. Each group included 10 implants placed in 10 separate bovine rib specimens.

### 2.6. Sample Size

The sample size was defined by the number of independent specimens and implants available for the original experiment. No formal a priori calculation specifically powered to detect the macrogeometry-by-technique interaction was performed. A retrospective post hoc power calculation was not used as a substitute for an a priori design calculation. Accordingly, the precision and magnitude of the observed effects were evaluated through 95% confidence intervals and partial eta squared values. The balanced allocation of 10 independent units per group allowed the planned 2 × 4 factorial analysis to be performed, although the sample size should be considered when interpreting subgroup comparisons and categorical outcomes.

### 2.7. Osteotomy Preparation

All osteotomies were prepared using a surgical motor operated at 800 rpm under continuous saline irrigation. The osteotomy depth was standardized for implants measuring 13 mm in length. The protocol parameters available from the original experimental record are reported below, including the final osteotomy diameters, osteotome sequence, and rotational direction used for osseodensification.

Four osteotomy preparation protocols were evaluated.

#### 2.7.1. Conventional Drilling

The conventional osteotomy was prepared using the progressive drilling sequence recommended by the implant manufacturer, with a final drill diameter of 3.5 mm.

#### 2.7.2. Undersized Drilling

The undersized drilling protocol followed a progressive preparation sequence, but the final osteotomy diameter was intentionally maintained below the nominal implant diameter.

The final drill diameter was 3.3 mm, resulting in an undersizing of 0.45 mm relative to the nominal 3.75 mm implant diameter.

#### 2.7.3. Bone Expansion

Bone expansion was performed using osteotomes of progressively increasing diameters. Osteotomes measuring 3.0, 3.5, and 4.0 mm were sequentially introduced to enlarge the osteotomy through lateral displacement and compaction of the surrounding trabecular bone.

The preparation was performed progressively to reduce the risk of specimen fracture and to obtain an osteotomy suitable for implant insertion.

#### 2.7.4. Osseodensification

Osseodensification was performed using densifying burs (Versah LLC, Jackson, MI, USA). The burs were operated in a counterclockwise direction at 800 rpm under continuous saline irrigation.

The goal of the protocol was to compact and displace the trabecular bone laterally during osteotomy preparation rather than to remove it primarily through conventional clockwise cutting.

### 2.8. Implant Placement

Immediately after completion of the assigned osteotomy protocol, the corresponding implant was inserted into the prepared site.

The same operator performed all implant insertions using a standardized clinical sequence, and each implant was advanced until its final planned position was reached. No single preset installation torque was imposed as an experimental input; insertion torque was recorded in N·cm as an observed mechanical outcome for each implant and is reported below. Only one implant was placed in each rib specimen.

No specimen was reused after implant removal, and no second osteotomy was performed on the same rib. This prevented the mechanical alteration of one experimental site from affecting another observation.

### 2.9. External Temperature Measurements

Temperature was recorded at three predefined procedural stages: before osteotomy preparation, immediately after completion of osteotomy preparation, and immediately after implant insertion.

Measurements were obtained using a digital infrared thermometer (Mestek, Cedar Rapids, IA, USA). The device was positioned at a standardized distance of 10 mm from the external surface of the osteotomy region.

Three consecutive temperature readings were obtained at each procedural stage, and their mean was used for statistical analysis.

The infrared thermometer measured external surface temperature and did not provide a direct measurement of internal temperature at the bone–implant interface. Temperature monitoring was included as a secondary procedural outcome to determine whether any preparation protocol was associated with a systematic externally detectable thermal change during osteotomy preparation or implant insertion; it was not intended to establish intraosseous thermal safety. Temperature was analyzed as a repeated outcome using a repeated-measures model with procedural stage as the within-unit factor and implant macrogeometry and osteotomy preparation technique as between-unit factors. Sphericity was assessed using Mauchly’s test; because this assumption was violated, Greenhouse–Geisser-corrected degrees of freedom were used for all effects involving procedural stage. The analysis was considered secondary and exploratory.

### 2.10. Immediate Mechanical Measurements

Insertion torque was recorded in N·cm for every implant during placement. These values were prospectively recorded in the original experimental dataset but were not reported in the initially submitted manuscript. For the present revision, they were incorporated as a complementary measure of the rotational resistance encountered during implant seating. No single fixed insertion-torque value was prescribed across experimental groups.

Resonance frequency analysis was also prospectively recorded for all 80 implants using an Osstell device (Osstell AB, Gothenburg, Sweden) and expressed as implant stability quotient (ISQ). ISQ was treated as a complementary measure of immediate implant stability and was analyzed separately from insertion and reverse torque because these measures reflect related but non-identical mechanical properties of the implant-bone system.

Reverse torque resistance was measured immediately after implant placement using a calibrated digital torque-measuring device (Kistler Instruments, Winterthur, Switzerland). It was defined as the rotational force required to initiate implant movement in the direction opposite to insertion, and values were recorded in N·cm. The procedure was performed by the same trained operator in all experimental groups.

Insertion torque, reverse torque resistance, and ISQ were analyzed using separate 2 × 4 factorial models with implant macrogeometry and osteotomy preparation technique as fixed between-unit factors and their interaction included in each model. Residual normality was assessed with the Shapiro–Wilk test and homogeneity of variance with Levene’s test. Because insertion torque and ISQ showed heteroscedasticity and ISQ residuals were non-normal, heteroscedasticity-consistent HC3 covariance estimates were used as sensitivity analyses. When an interaction was significant, simple effects were examined using Welch comparisons with Holm adjustment where variance assumptions were not satisfied; otherwise, marginal technique comparisons were evaluated with Welch tests and Holm adjustment. Mean differences are reported with 95% confidence intervals, and partial eta squared was used as the ANOVA effect-size measure. Pearson correlations were calculated among insertion torque, reverse torque resistance, ISQ, and Periotest values, with Spearman rank correlations used as sensitivity analyses. Periotest was analyzed as a secondary complementary outcome using the same factorial structure. Reverse torque, insertion torque, ISQ, and Periotest were not considered interchangeable or validated clinical predictors of osseointegration or implant survival [4,5].

### 2.11. Implant Removal

After the reverse torque value was recorded, the implant was completely removed by continuing controlled rotation in the reverse direction.

The implants were removed through rotational disengagement without levering, bending, or directly deforming the implant body. After removal, each implant was handled individually to avoid contact between specimens and was stored separately until morphological assessment.

Because the implants were examined after reverse torque testing and complete removal, the visually detected thread alterations cannot be attributed exclusively to the insertion phase. They may reflect mechanical events occurring during implant insertion, reverse torque application, removal, or a combination of these procedures.

### 2.12. Assessment of Regional Thread Alterations

After removal, each implant was examined under a stereomicroscope at 20× magnification (Leica MZ6; Leica Microsystems, Wetzlar, Germany).

For regional assessment, each implant was divided a priori into the cervical region (including the cervical microthreads), the body region (middle portion), and the apical region.

Thread alteration was defined operationally as a visually detectable change in the expected thread contour, including flattening, bending, displacement, notching, loss of continuity, or another clearly discernible contour modification. The term describes a post-removal visual finding and does not establish plastic deformation, material loss, surface abrasion, or functional damage.

For each region, alterations were recorded as either present or absent. The assessment therefore generated a binary qualitative outcome and did not quantify the magnitude, depth, area, or volume of the observed alteration.

Two independent examiners evaluated the implants without access to the osteotomy preparation protocol. Complete blinding to implant macrogeometry was not possible because the V2 and Quattro designs are visually distinguishable. Disagreements were resolved by joint reassessment and consensus. The initial examiner-specific classifications were not retained in a format that permitted retrospective calculation of interobserver agreement. Paired pre-insertion images of each individual implant were not acquired as part of the original protocol; consequently, no paired before-and-after image analysis can be provided retrospectively.

The assessment was intentionally qualitative. No scanning electron microscopy, energy-dispersive X-ray spectroscopy, surface profilometry, quantitative morphometry, micro-computed tomography, or fractal analysis was included in the present investigation. Thread alterations were analyzed separately for the cervical, body, and apical regions using contingency tables. Associations with implant macrogeometry were evaluated using Fisher’s exact test when expected cell frequencies were small; associations with the four-level osteotomy preparation factor were evaluated using the Fisher–Freeman–Halton exact test when Pearson chi-square assumptions were not satisfied. Pairwise comparisons between preparation techniques used two-sided Fisher’s exact tests with Holm adjustment. No inferential test was performed for the implant body because no alterations occurred in that region.

### 2.13. Data Management

The individual implant–rib combination was used as the experimental unit. The database contained one record for each of the 80 independent experimental units.

Before statistical analysis, categorical variables were standardized to ensure consistent coding of the implant macrogeometry and the osteotomy preparation technique. Data were checked for missing values, implausible values, coding errors, and inconsistencies between the original database and the summary tables.

No missing observations were identified for the principal study outcomes.

ChatGPTChatGPT powered by the GPT-5.6 Sol model (OpenAI; accessed on 8 September 2026) was used to assist with language refinement and figure formatting; no study data were generated or modified using the tool. The original study analyses were performed using IBM SPSS Statistics, version 26.0 (IBM Corp., Armonk, NY, USA). For this revision, the prospectively recorded insertion torque, ISQ, and Periotest variables and the cross-measure correlations were independently reanalyzed in Python 3.13.5 using SciPy 1.17.0 and statsmodels 0.14.6. All numerical outputs were independently checked against the original dataset by the authors. Continuous variables are summarized as means and standard deviations, categorical variables as absolute frequencies and proportions, all tests were two-sided, and statistical significance was established at *p* < 0.05.

## 3. Results

### 3.1. Experimental Sample

A total of 80 implants were included in the analysis. The implants were equally distributed among the eight experimental groups generated by the combination of two implant macrogeometries and four osteotomy preparation techniques. Each group comprised 10 independent implant–rib experimental units.

All implants were successfully placed. Insertion torque, reverse torque resistance, ISQ, and Periotest values were available for all 80 experimental units; all implants were subsequently removed and examined. No missing values were identified for the principal mechanical or structural outcomes.

### 3.2. Immediate Mechanical Outcomes

Insertion torque, reverse torque resistance, and ISQ are summarized together in Table 1 to allow direct comparison of the three immediate mechanical measures across the eight experimental groups.

#### 3.2.1. Insertion Torque

Insertion-torque residuals were compatible with normality (Shapiro–Wilk *p* = 0.436), whereas Levene’s test indicated heterogeneity of variance among the eight groups (*p* = 0.022). The factorial ANOVA showed significant effects of implant macrogeometry (F(1,72) = 29.69, *p* < 0.001; partial η^2^ = 0.292) and osteotomy preparation technique (F(3,72) = 5.26, *p* = 0.002; partial η^2^ = 0.180), but no macrogeometry-by-technique interaction (F(3,72) = 1.21, *p* = 0.314; partial η^2^ = 0.048). HC3 sensitivity analysis confirmed the macrogeometry effect (*p* < 0.001), the technique effect (*p* = 0.007), and the absence of interaction (*p* = 0.334).

Across implant designs, undersized drilling produced greater insertion torque than conventional drilling (mean difference 10.5 N·cm; 95% CI 3.0 to 18.0; Holm-adjusted *p* = 0.044) and osseodensification (mean difference 7.85 N·cm; 95% CI 2.16 to 13.54; adjusted *p* = 0.044). The remaining technique comparisons were not significant after Holm adjustment.

#### 3.2.2. Reverse Torque Resistance

Reverse-torque residuals did not significantly deviate from normality (Shapiro–Wilk *p* = 0.210), and Levene’s test did not indicate heterogeneity of variance (*p* = 0.354). Two-way ANOVA identified significant effects of implant macrogeometry (F(1,72) = 52.00, *p* < 0.001; partial η^2^ = 0.419), osteotomy preparation technique (F(3,72) = 9.01, *p* < 0.001; partial η^2^ = 0.273), and their interaction (F(3,72) = 7.72, *p* < 0.001; partial η^2^ = 0.243). Because the interaction was significant, simple effects were interpreted rather than the main effects in isolation.

Within the V2 design, compared with conventional drilling, osseodensification resulted in significantly greater reverse torque resistance, with a mean difference of 21.9 N·cm (95% confidence interval (CI): 9.8 to 34.0; *p* < 0.001).

Osseodensification also resulted in greater reverse torque resistance than undersized drilling did, with a mean difference of 15.0 N·cm (95% CI: 2.9 to 27.1; *p* = 0.009), and greater resistance than bone expansion did, with a mean difference of 17.1 N·cm (95% CI: 5.0 to 29.2; *p* = 0.002).

No statistically significant differences were observed among conventional drilling, undersized drilling, and bone expansion within the V2 design.

Within the Quattro design, undersized drilling resulted in significantly greater reverse torque resistance than conventional drilling did, with a mean difference of 19.3 N·cm (95% CI: 7.2 to 31.4; *p* < 0.001).

Compared with conventional drilling, bone expansion also resulted in greater reverse torque resistance, with a mean difference of 19.3 N·cm (95% CI: 7.2 to 31.4; *p* < 0.001).

#### 3.2.3. Comparisons Between Implant Designs Within Each Preparation Technique

After conventional drilling, the mean reverse torque resistance of the Quattro implants was 13.1 N·cm greater than that of the V2 implants (95% CI: 4.0 to 22.2; Holm-adjusted *p* = 0.011).

After undersized drilling, the Quattro design exceeded the V2 design by 25.5 N·cm (95% CI: 16.4 to 34.6; adjusted *p* < 0.001).

After bone expansion, the difference between the Quattro and V2 designs was 27.6 N·cm (95% CI: 18.5 to 36.7; adjusted *p* < 0.001).

No difference was observed between implant designs after osseodensification. Both designs presented a mean reverse torque resistance of 39.0 N·cm, with a mean difference of 0.0 N·cm (95% CI: −9.1 to 9.1; *p* = 1.000).

The distribution of reverse torque resistance across the eight experimental groups is illustrated in Figure 2.

#### 3.2.4. Implant Stability Quotient (ISQ)

ISQ residuals departed from normality (Shapiro–Wilk *p* < 0.001), and Levene’s test indicated heterogeneous variances (*p* = 0.012). The conventional factorial ANOVA showed a significant macrogeometry effect (F(1,72) = 29.55, *p* < 0.001; partial η^2^ = 0.291), no overall technique effect (F(3,72) = 1.85, *p* = 0.146; partial η^2^ = 0.072), and a significant macrogeometry-by-technique interaction (F(3,72) = 3.09, *p* = 0.032; partial η^2^ = 0.114). HC3 sensitivity analysis confirmed the macrogeometry effect (*p* < 0.001), no overall technique effect (*p* = 0.235), and the interaction (*p* = 0.0037).

Holm-adjusted Welch comparisons showed that the Quattro design had higher ISQ than V2 after bone expansion (mean difference 9.8 ISQ units; 95% CI 6.65 to 12.95; adjusted *p* < 0.001), whereas between-design differences after conventional drilling, undersized drilling, and osseodensification were not significant after adjustment. Within V2, undersized drilling produced higher ISQ than bone expansion (mean difference 5.0; 95% CI 1.55 to 8.45; adjusted *p* = 0.043). Within Quattro, bone expansion produced higher ISQ than osseodensification (mean difference 3.9; 95% CI 2.15 to 5.65; adjusted *p* = 0.001).

#### 3.2.5. Relationships Among Mechanical Measures and Periotest

Insertion torque correlated strongly with reverse torque resistance (Pearson r = 0.645, *p* < 0.001; Spearman ρ = 0.628, *p* < 0.001). ISQ showed a more moderate association with reverse torque resistance (r = 0.370, *p* < 0.001; ρ = 0.510, *p* < 0.001) and a moderate association with insertion torque (r = 0.496, *p* < 0.001; ρ = 0.599, *p* < 0.001). Periotest values were inversely associated with reverse torque resistance (r = −0.373, *p* < 0.001), insertion torque (r = −0.436, *p* < 0.001), and ISQ (r = −0.617, *p* < 0.001). In the factorial Periotest analysis, macrogeometry was significant (F(1,72) = 15.74, *p* < 0.001; partial η^2^ = 0.179), whereas technique (*p* = 0.507) and the interaction (*p* = 0.401) were not; HC3 sensitivity analyses yielded the same inferential pattern. These findings support convergent but non-interchangeable information from the different immediate mechanical measurements.

### 3.3. External Temperature

External surface temperature was recorded at baseline, immediately after osteotomy preparation, and immediately after implant insertion. Descriptive values for the eight experimental groups are presented in Table 2.

Across all the experimental units, the mean temperatures were 30.67 ± 2.49 °C at baseline, 30.56 ± 2.10 °C after osteotomy preparation, and 30.61 ± 2.27 °C after implant insertion. The recorded values ranged from 21.4 to 34.4 °C.

Mauchly’s test indicated that the assumption of sphericity was violated; therefore, Greenhouse–Geisser-corrected results are reported. The repeated-measures analysis revealed no overall change in external temperature across the three procedural stages (F(1.75, 126.13) = 0.07, *p* = 0.912; partial η^2^ = 0.001).

No significant stage-by-macrogeometry interaction was observed (F(1.75, 126.13) = 0.15, *p* = 0.833; partial η^2^ = 0.002), and the stage-by-preparation technique interaction was also not significant (F(5.26, 126.13) = 0.62, *p* = 0.693; partial η^2^ = 0.025).

The three-way interaction between procedural stage, implant macrogeometry, and preparation technique remained statistically significant after Greenhouse–Geisser correction (F(5.26, 126.13) = 2.45, *p* = 0.035; partial η^2^ = 0.093). This interaction reflected small and nonuniform temperature changes among individual experimental combinations rather than a consistent increase associated with a particular technique.

For example, after conventional drilling, the external temperature decreased by a mean of 1.45 °C in the Quattro group but increased by 1.51 °C in the V2 group. Following bone expansion, the temperature increased by 1.12 °C in the Quattro group but decreased by 1.02 °C in the V2 group.

The changes from baseline to after implant insertion were small across all groups. Neither the implant macrogeometry nor the preparation technique resulted in a consistent increase in the externally measured temperature.

Because these values were obtained by infrared surface thermometry, they represent external procedural temperatures and not direct measurements at the internal bone–implant interface.

### 3.4. Regional Thread Alterations

#### 3.4.1. Cervical Region

The regional distribution of visually detectable thread alterations according to the implant macrogeometry and osteotomy preparation technique is presented in Table 3.

Alterations were observed in 6 of the 40 V2 implants and 8 of the 40 Quattro implants. No significant association was detected between the implant macrogeometry and cervical thread alterations (Fisher’s exact test, *p* = 0.770).

Cervical alterations occurred in 0 of 20 implants after conventional drilling, 12 of 20 after undersized drilling, 0 of 20 after bone expansion, and 2 of 20 after osseodensification.

A significant overall association was observed between the osteotomy preparation technique and cervical thread alterations (Fisher–Freeman–Halton exact test, *p* < 0.001).

Adjusted pairwise analyses revealed that cervical alterations were significantly more frequent after undersized drilling than after conventional drilling (Holm-adjusted *p* < 0.001), bone expansion (adjusted *p* < 0.001), or osseodensification (adjusted *p* = 0.009).

No significant differences were detected among conventional drilling, bone expansion, and osseodensification.

These findings indicate that cervical thread alteration was primarily associated with the undersized drilling protocol rather than with implant macrogeometry.

#### 3.4.2. Body Region

No visually detectable thread alterations were observed in the implant body in any of the 80 implants.

Because this outcome showed no variability, no inferential statistical analysis was performed for the implant body region.

#### 3.4.3. Apical Region

Apical thread alterations were identified in 37 of the 80 implants.

None of the 40 V2 implants presented an apical alteration, whereas alterations were detected in 37 of the 40 Quattro implants. The association between implant macrogeometry and apical thread alteration was statistically significant (Fisher’s exact test, *p* < 0.001).

Apical alterations occurred in 10 of 20 implants after conventional drilling, 8 of 20 after undersized drilling, 9 of 20 after bone expansion, and 10 of 20 after osseodensification.

No significant association was found between the preparation technique and apical thread alterations (χ^2^(3) = 0.55, *p* = 0.907).

Thus, apical thread alterations were strongly associated with the Quattro macrogeometry and were not detectably influenced by the osteotomy preparation technique. The regional distribution of cervical and apical thread alterations is illustrated in Figure 3.

## 4. Discussion

The principal finding of this ex vivo study was that implant macrogeometry and osteotomy preparation technique did not act as independent determinants of immediate resistance to rotational disengagement. A significant interaction between both factors was observed for reverse torque resistance, indicating that the mechanical response to a given preparation protocol depended on the implant design. This interpretation is restricted to the experimental reverse-torque outcome and should not be extrapolated directly to clinical osseointegration or survival.

In the V2 design, compared with conventional drilling, undersized drilling, and bone expansion, osseodensification resulted in significantly greater reverse torque resistance. In contrast, in the Quattro design, undersized drilling and bone expansion resulted in significantly greater reverse torque resistance than conventional drilling did, whereas osseodensification did not result in an additional increase. These findings support the concept that implant design and osteotomy preparation should be considered together when interpreting immediate implant mechanics [2,6].

The interaction observed in the present experiment is biomechanically plausible. Implant shape, thread depth, pitch, flank geometry, cutting characteristics, and cervical microthreads determine the pattern of contact between the implant and osteotomy walls and influence the transmission of insertion-related forces [2,18,19,20,21,22]. Recent experimental and review evidence confirms that macrogeometry can substantially modify insertion torque, removal torque, and resonance-frequency measurements, especially in low-density substrates [19,20,21,22]. In the present study, the Quattro design generated higher reverse torque resistance than the V2 design after conventional drilling, undersized drilling, and bone expansion, but not after osseodensification. The convergence of both designs at a mean reverse torque resistance of 39.0 N·cm after osseodensification indicates that the mechanical effect of this protocol was macrogeometry-dependent rather than uniformly additive.

The greater reverse torque resistance observed with undersized drilling and bone expansion in Quattro implants is consistent with the mechanical principle that reducing or progressively compacting the osteotomy can increase interference between implant threads and the surrounding substrate. Evidence syntheses indicate that underpreparation and osteotome-based site preparation can increase surrogate measures of primary stability in low-density bone, although the biological and clinical consequences depend on bone density and the degree of implant-to-osteotomy mismatch [7,8,26]. Falisi et al. demonstrated that both surgical preparation and implant macrogeometry affected primary stability in an in vitro model [6]. Romeo et al. also showed that insertion and removal torque values varied according to drilling protocol and artificial bone density [25]. These studies support reverse or removal torque as comparative experimental variables, but not as validated predictors of clinical osseointegration or long-term success.

Osseodensification is intended to preserve and laterally compact trabecular bone rather than remove it primarily through conventional cutting. Experimental evidence has demonstrated increased insertion torque, implant stability, and bone-to-implant contact after osseodensification in low-density models [9,13]. Systematic reviews and meta-analyses published between 2022 and 2026 generally report higher immediate stability measures with osseodensification than with conventional drilling in low-density bone, but they also emphasize heterogeneity, variable certainty of evidence, and a lack of demonstrated long-term superiority for survival or marginal bone preservation [8,11,12,14,15,16]. These qualifications are important when translating an ex vivo mechanical finding to clinical practice.

The present results add important information to this evidence. Osseodensification did not produce the same relative effect in both implant macrogeometries. It substantially increased reverse torque resistance in V2 implants but did not exceed the values obtained with undersized drilling or bone expansion in Quattro implants. Therefore, the mechanical effect of osseodensification should not be assumed to be independent of the implant design.

The incorporation of the prospectively recorded insertion-torque and ISQ data provides an important triangulation of the reverse-torque findings. Insertion torque showed significant independent effects of macrogeometry and preparation technique but no interaction, whereas ISQ showed a significant macrogeometry-by-technique interaction, particularly because Quattro implants achieved higher ISQ after bone expansion. Reverse torque therefore did not simply reproduce insertion torque or ISQ. The strong association between insertion and reverse torque (r = 0.645), together with the more moderate association between ISQ and reverse torque (r = 0.370), supports the interpretation that these measurements characterize related but distinct components of immediate mechanical fixation [1,2,3,4,5]. This complementary pattern strengthens the biomechanical interpretation while simultaneously arguing against reliance on any single surrogate stability metric as a clinical threshold.

Periotest provided additional convergent information: values differed significantly by macrogeometry and were inversely associated with insertion torque, reverse torque, and ISQ, but neither preparation technique nor the macrogeometry-by-technique interaction was significant. Because Periotest was not a principal study outcome and adds less information to the specific interaction hypothesis, it is reported as a complementary secondary analysis rather than given the same emphasis as insertion torque, reverse torque, and ISQ.

A second relevant finding was the distinct regional distribution of visually detectable thread alterations. Cervical alterations were strongly associated with undersized drilling. They were observed in 12 of 20 implants prepared using this protocol, compared with none after conventional drilling or bone expansion and two after osseodensification. Conversely, cervical alterations were not significantly associated with implant macrogeometry.

This pattern suggests that the reduced osteotomy diameter generated mechanical conditions capable of modifying the cervical thread contour during insertion, reverse torque testing, removal, or a combination of these stages. The final osteotomy diameter in the undersized protocol was 0.45 mm smaller than the nominal implant diameter. Increased mechanical interference and radial compression therefore represent a plausible explanation. However, the local strain, pressure, and stress distribution were not directly measured, and this mechanism must be regarded as an interpretation rather than a demonstrated causal pathway.

Apical alterations followed a markedly different pattern. They were absent in all 40 V2 implants but were detected in 37 of the 40 Quattro implants. No significant association was observed between apical alterations and the osteotomy preparation technique. These findings indicate that the apical pattern was predominantly associated with the Quattro macrogeometry under the present experimental conditions.

The Quattro design incorporates a deeper and more pronounced thread configuration in its body and apical regions. Such characteristics may have resulted in greater localized engagement during insertion or removal. Contemporary experimental work and reviews show that thread geometry and macrodesign influence local mechanical engagement and primary stability measures [19,20,21,22]. Silva et al. also demonstrated that macrogeometry and bone type affected insertion torque, primary stability, surface topography changes, and titanium release during implant insertion into artificial bone [23].

Nevertheless, the findings of Silva et al. should not be interpreted as direct confirmation of the alterations observed in the present study. Their study used artificial bone, quantitative surface analysis, and different implant designs. This finding supports only the broader concept that insertion-related surface changes may depend on macrogeometry and substrate properties [23].

Gehrke et al. reported that high applied torque could produce structural modifications in implant connections [24]. That investigation assessed implant connection components rather than external threads and used torque values and testing conditions that were different from those of the present study. It therefore supports the general possibility of torque-related implant alteration but does not establish that our apical findings represent equivalent damage or have the same mechanical implications.

No alterations were detected in the implant body. The regional specificity of the observations—cervical alterations associated mainly with undersized drilling and apical alterations associated mainly with the Quattro design—argues against a uniform process affecting the entire implant. Instead, these findings suggest that visually detectable changes were concentrated in regions where local macrogeometry and the mechanical constraints of the preparation protocol interacted most strongly.

However, the implants were evaluated only after reverse torque testing and complete removal. It is therefore impossible to determine whether the observed alterations arose during insertion, at the initiation of reverse rotation, during complete removal, or cumulatively across these stages. For this reason, the findings are described as post-removal regional thread alterations rather than insertion-induced structural damage.

The qualitative assessment of thread morphology represents both the principal exploratory contribution and an important methodological limitation. Examination at predefined cervical, body, and apical regions allowed regional patterns to be identified, but binary classification as present or absent did not quantify the depth, area, volume, or severity of the alterations. It also could not distinguish with certainty among plastic deformation, surface abrasion, compacted bone debris, titanium galling, loss of surface material, notching, or other types of morphological change. Although two independent examiners were blinded to preparation technique, complete blinding to macrogeometry was impossible because the designs were visually distinguishable, and interobserver agreement could not be calculated from the retained records. In addition, paired pre-insertion images of each implant were not available.

Accordingly, no inference can be made regarding fatigue resistance, fracture risk, osseointegration, marginal bone maintenance, implant survival, or prosthetic performance. Quantitative surface characterization using scanning electron microscopy, energy-dispersive X-ray spectroscopy, profilometry, micro-computed tomography, or validated morphometric methods would be necessary to determine the physical nature and magnitude of the observed alterations. Such analyses were not part of the original protocol and cannot be reconstructed reliably from the present dataset; they should be incorporated prospectively in future experiments.

External surface temperature remained broadly stable throughout the procedure. There was no overall change from baseline to osteotomy preparation and implant insertion, and neither macrogeometry nor preparation technique produced a consistent externally measured increase. Infrared thermometry and thermography have been used experimentally to compare externally detected temperature changes during implant-site preparation [27,28,29,30]. Recent studies confirm that drilling protocol, bone density, irrigation, and drill sequence can influence externally measured temperature; however, surface thermography is not a direct substitute for intraosseous thermocouple measurements [28,29,30].

The statistically significant three-way interaction observed for temperature reflected small and nonuniform changes among particular experimental combinations rather than a consistent thermal increase associated with one technique. Because infrared thermometry measures radiation from the external surface, the present findings should be interpreted only as showing that no systematic increase was detected at the measured surface. They do not demonstrate that temperature at the internal bone–implant interface remained below a biological injury threshold, and the manuscript therefore makes no claim of thermal safety.

The bovine rib model provided independent biological specimens and preserved the trabecular architecture and biological heterogeneity that could not be fully replicated by homogeneous synthetic blocks. Before the definitive experiment, bovine vertebrae, rabbit tibiae, porcine ribs, and bovine ribs were preliminarily assessed. Bovine ribs were selected because their tactile response during preparation was considered by the operating surgeon to resemble that experienced clinically in low-density maxillary bone.

Nevertheless, model selection was subjective and based on one operator. The specimens were not characterized by computed tomography, histomorphometry, cortical-thickness measurement, density calibration, or mechanical testing. Consequently, they cannot be objectively classified as human type III or IV bone. Contemporary evidence shows that radiographic bone density, cortical thickness, substrate properties, and local bone support are associated with primary stability measures [3,31,32]. A recent low-density bovine-rib experiment incorporated micro-computed tomography before implant placement, illustrating an objective structural approach that should be adopted in future work [33]. Artificial bone would have provided greater standardization, whereas bovine ribs provided biological architecture and heterogeneity; this trade-off should be considered when interpreting the present findings [23,25,31,33].

This study has several strengths. It used a balanced factorial design, two implants with identical nominal dimensions and surface treatments, one independent rib specimen per implant, a single experienced operator, and a complete dataset comprising 80 experimental units. The revised analysis triangulates immediate mechanical behavior using insertion torque, reverse torque resistance, ISQ, and a complementary Periotest analysis rather than relying on reverse torque in isolation. The statistical analysis explicitly evaluated macrogeometry-by-technique interactions, effect sizes, confidence intervals, multiplicity-adjusted comparisons, and robust sensitivity analyses when distributional assumptions were not satisfied. The regional evaluation also distinguished cervical, body, and apical patterns rather than combining all implant alterations into a single outcome.

From a biomechanical perspective, the findings indicate that selecting an osteotomy protocol without considering implant macrogeometry may result in a different immediate mechanical response from that anticipated. The results do not support recommending one preparation technique universally. Instead, implant design and osteotomy preparation should be viewed as components of a combined mechanical system. Importantly, contemporary systematic reviews also caution against treating a single insertion-torque or ISQ threshold as an independent predictor of implant survival [4,5]; the present reverse-torque findings should therefore be interpreted as experimental, hypothesis-generating evidence rather than a clinical decision threshold.

In particular, undersized drilling was associated with cervical thread alterations, whereas the Quattro macrogeometry was associated with apical alterations irrespective of the preparation technique. These observations should be regarded as hypothesis-generating until confirmed using objectively characterized bone density models, pre- and postinsertion quantitative imaging, surface characterization, and mechanical testing designed to determine whether the alterations have functional consequences.

### Limitations

Several limitations should be considered when interpreting the present findings. First, this was an ex vivo study using bovine rib specimens and therefore could not reproduce the biological complexity of living human bone, including vascularization, remodeling, healing, and patient-related variability. Although bovine ribs were selected after preliminary tactile comparison with other animal-derived substrates, this selection was based on the subjective assessment of a single experienced surgeon. Bone density and cortical thickness were not objectively characterized by micro-computed tomography, histomorphometry, calibrated radiodensity, indentation testing, or another mechanical method; therefore, the specimens cannot formally be classified as human type III or IV bone [3,31,32,33].

Second, no formal a priori sample-size calculation was specifically performed for the macrogeometry-by-technique interaction. A retrospective post hoc power calculation was not used to replace this missing design step. Although the balanced design enabled factorial analysis and several observed effects were large, the study may have had limited precision for subgroup comparisons and categorical outcomes; effect sizes and 95% confidence intervals should therefore be considered together with *p* values.

Third, reverse torque resistance was measured immediately after implant placement and remained the primary mechanical endpoint. Insertion torque and ISQ were prospectively recorded in the original dataset and are now incorporated as complementary outcomes, with Periotest analyzed secondarily. Although these measures provide convergent biomechanical information, none is interchangeable with another, and none independently represents micromotion, biological fixation, clinical osseointegration, or a validated predictor of implant survival [4,5].

Fourth, thread alterations were evaluated qualitatively as present or absent in predefined cervical, body, and apical regions. This method identified regional patterns but did not quantify their magnitude, area, depth, volume, composition, or severity and could not distinguish plastic deformation from abrasion, bone-debris compaction, titanium galling, or other surface phenomena. Two independent examiners were blinded to the osteotomy preparation protocol, but complete blinding to macrogeometry was impossible because the designs were visually distinguishable; interobserver agreement could not be quantified because the initial examiner-specific classifications were not retained. Paired pre-insertion images of each implant were also not available, and no SEM, EDS, profilometry, or quantitative morphometry was performed.

Fifth, the implants were examined only after reverse torque testing and complete removal. Therefore, the observed alterations cannot be attributed exclusively to forward insertion and may have occurred during insertion, initiation of reverse rotation, complete removal, or cumulatively across these stages. The findings are therefore described as post-removal thread alterations rather than insertion-induced damage or deformation.

Sixth, only two implant macrogeometries from a single manufacturer were evaluated. The findings should not be generalized to other implant systems, dimensions, materials, thread designs, or surface characteristics. In addition, the original experimental record did not include every intermediate instrument dimension required to reproduce all four osteotomy sequences beyond the parameters reported in the Methods; no unrecorded procedural detail has been retrospectively inferred.

Finally, temperature was measured by external infrared thermometry. The recorded values represented surface temperature and did not directly measure thermal changes at the internal bone–implant interface. Consequently, the temperature analysis is a secondary procedural comparison and cannot establish intraosseous thermal safety [28,29,30].

## 5. Conclusions

Within the limitations of this ex vivo biomechanical study, implant macrogeometry and osteotomy preparation technique interacted significantly in determining immediate reverse torque resistance and ISQ, whereas insertion torque showed significant independent effects of macrogeometry and preparation technique without a significant interaction.

The complementary mechanical outcomes were related but non-interchangeable. Insertion torque correlated strongly with reverse torque resistance, while ISQ showed a more moderate association. Osseodensification produced the greatest reverse torque resistance in V2 implants, whereas undersized drilling and bone expansion produced the greatest reverse-torque values in Quattro implants; for ISQ, the clearest between-design difference occurred after bone expansion, favoring Quattro.

Visually detectable thread alterations showed distinct regional patterns. Cervical alterations were primarily associated with undersized drilling, whereas apical alterations were strongly associated with the Quattro macrogeometry and were not significantly influenced by the preparation technique. No alterations were detected in the implant body.

These findings indicate that implant macrogeometry and osteotomy preparation should be considered as an interdependent biomechanical system rather than as isolated determinants of immediate fixation. However, insertion torque, reverse torque, ISQ, and Periotest remain surrogate mechanical measurements and should not be equated with clinical osseointegration or long-term implant outcome. The physical nature and functional or clinical significance of the visually detectable post-removal thread alterations also remain unknown and require confirmation through prospectively powered studies using objectively characterized bone models, paired pre- and post-insertion imaging, quantitative surface characterization, and ultimately in vivo or clinical investigations.

## Figures and Tables

**Figure 1:**
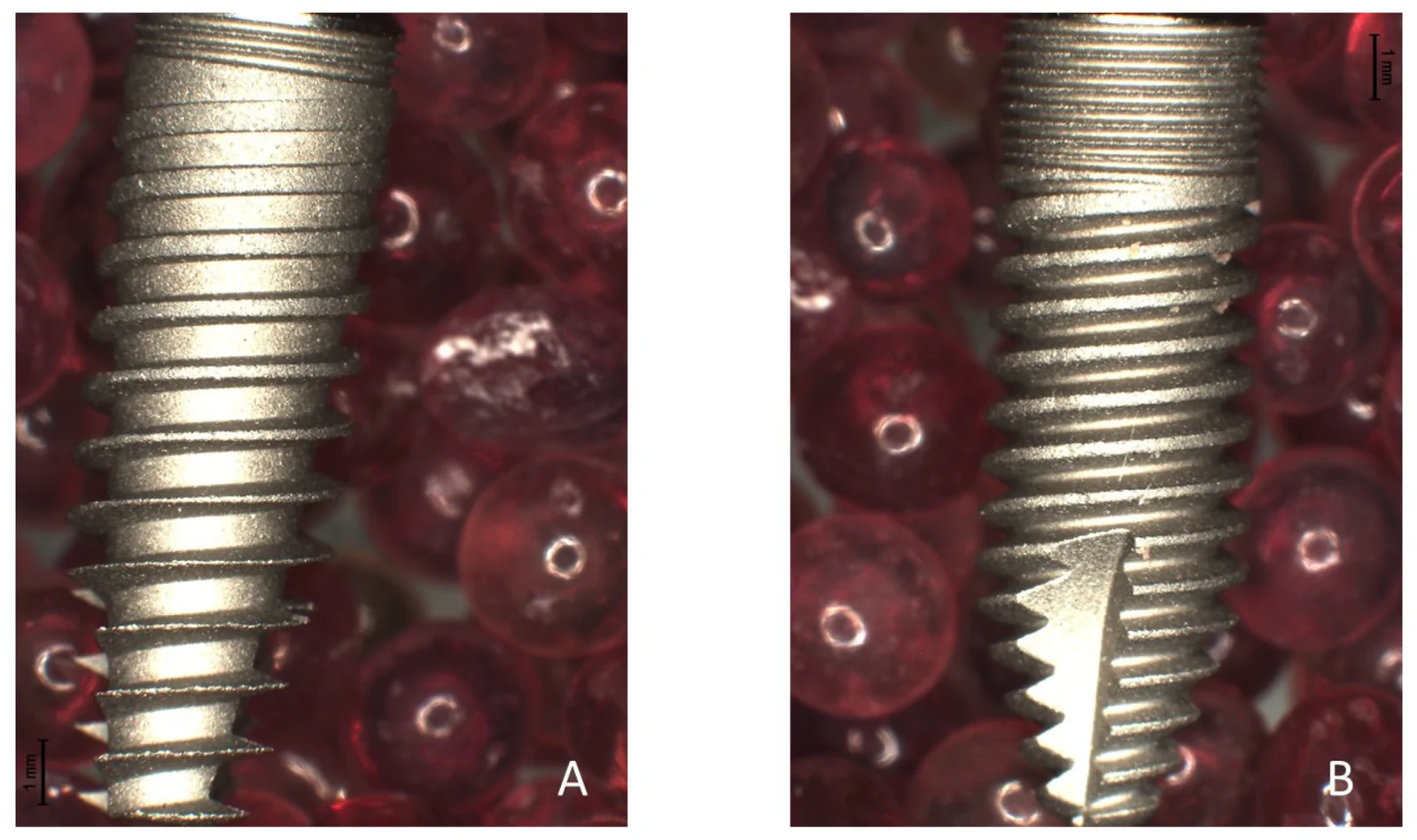
Representative stereomicroscopy images of the two implant macrogeometries evaluated: (**A**) V2 design and (**B**) Quattro design. Scale bars = 1 mm.

**Figure 2:**
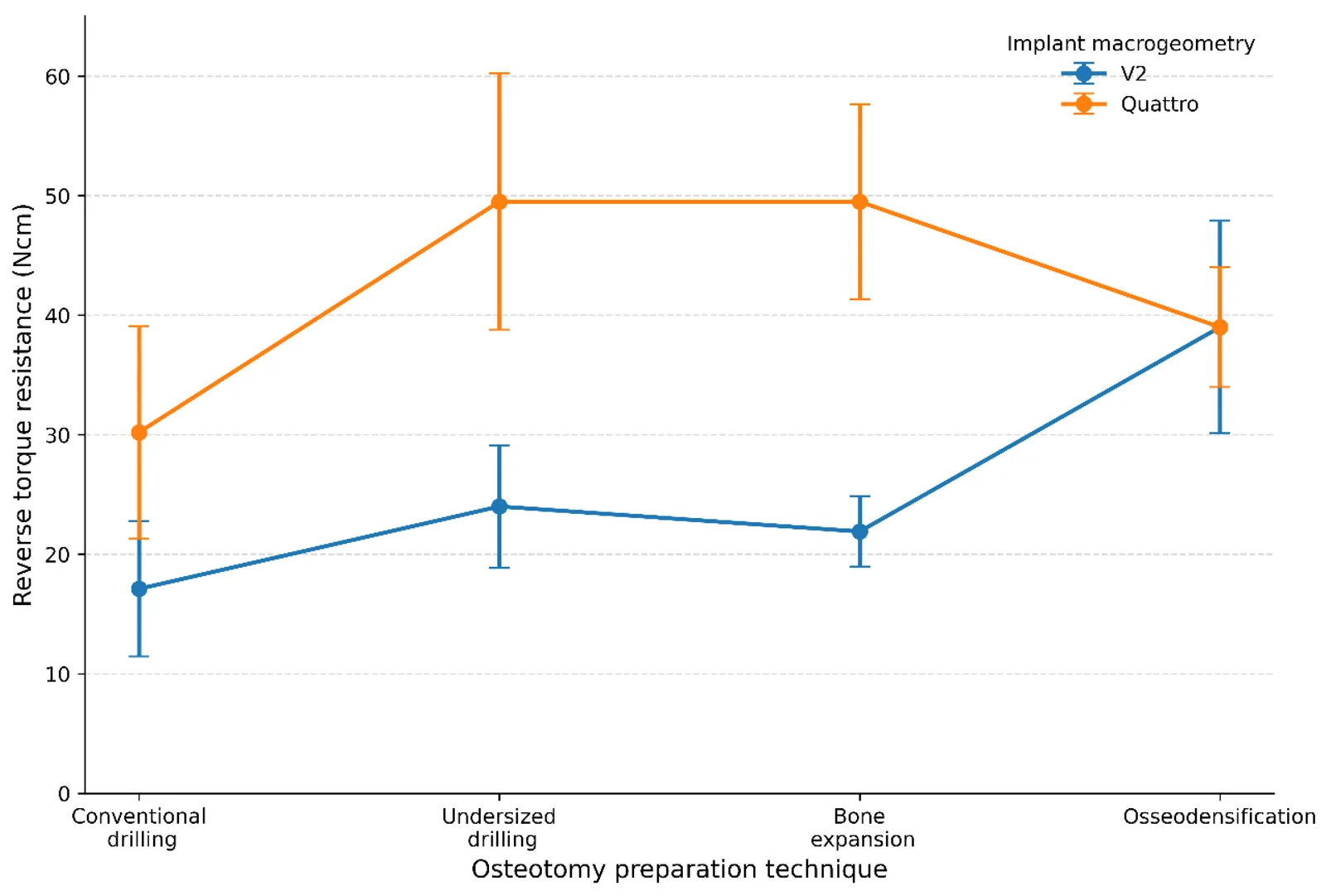
Reverse torque resistance according to the implant macrogeometry and osteotomy preparation technique. Data are presented as the means with 95% confidence intervals.

**Figure 3:**
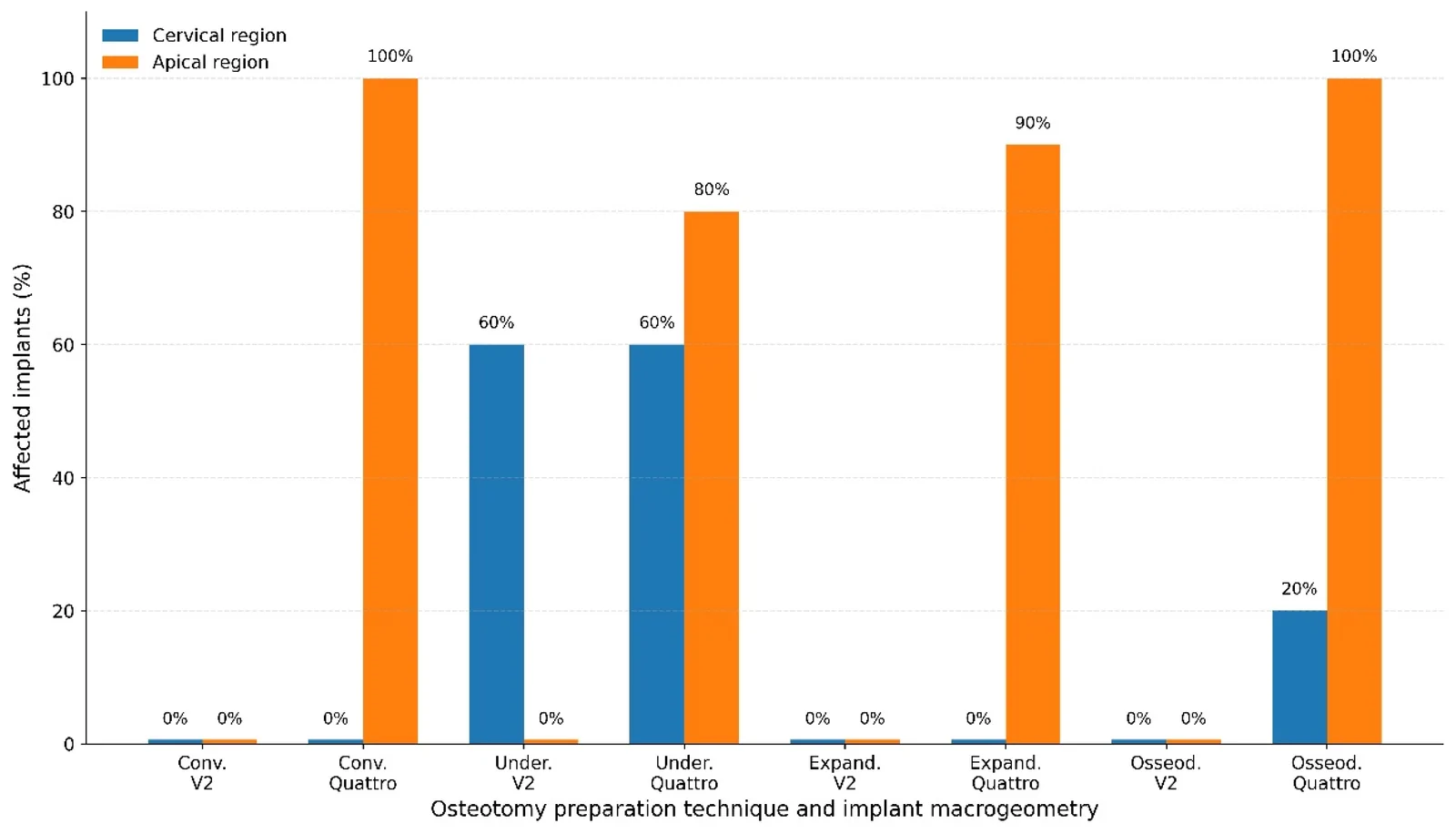
Cervical and apical thread alterations according to the implant macrogeometry and osteotomy preparation technique. The values are the percentages of affected implants. Abbreviations: Conv., conventional; Under., undersized; Expand., expansion; Osseod., osseodensification.

**Table 1:** Insertion torque, reverse torque resistance, and implant stability quotient according to implant macrogeometry and osteotomy preparation technique.

Implant Macrogeometry	Osteotomy Preparation Technique	Insertion Torque, N·cm	Reverse Torque, N·cm	ISQ
V2	Conventional drilling	23.0 ± 10.1	17.1 ± 7.9	72.4 ± 3.1
V2	Undersized drilling	36.0 ± 7.4	24.0 ± 7.1	72.8 ± 3.0
V2	Bone expansion	28.5 ± 3.4	21.9 ± 4.1	67.8 ± 4.2
V2	Osseodensification	30.5 ± 10.7	39.0 ± 12.4	69.6 ± 8.4
Quattro	Conventional drilling	37.7 ± 12.9	30.2 ± 12.4	76.6 ± 4.5
Quattro	Undersized drilling	45.7 ± 8.9	49.5 ± 15.0	75.0 ± 2.5
Quattro	Bone expansion	41.5 ± 7.8	49.5 ± 11.4	77.6 ± 1.9
Quattro	Osseodensification	35.5 ± 4.4	39.0 ± 7.0	73.7 ± 1.8

Data are presented as mean ± standard deviation; *n* = 10 implants per experimental group. Insertion torque and reverse torque are expressed in N·cm; ISQ is dimensionless.

**Table 2:** External surface temperature recorded at the three procedural stages according to the implant macrogeometry and osteotomy preparation technique.

Implant Macrogeometry	Osteotomy Preparation Technique	Baseline, °C	After Osteotomy Preparation, °C	After Implant Insertion, °C
V2	Conventional drilling	29.66 ± 3.05	31.17 ± 2.53	29.83 ± 3.05
V2	Undersized drilling	31.19 ± 2.91	30.33 ± 2.07	31.57 ± 1.97
V2	Bone expansion	32.23 ± 1.23	31.21 ± 1.77	31.08 ± 1.59
V2	Osseodensification	30.97 ± 2.10	30.60 ± 2.11	30.65 ± 2.30
Quattro	Conventional drilling	32.02 ± 1.80	30.57 ± 2.26	31.19 ± 2.26
Quattro	Undersized drilling	30.05 ± 2.35	30.09 ± 2.12	30.23 ± 2.40
Quattro	Bone expansion	28.68 ± 2.96	29.80 ± 1.86	30.83 ± 1.97
Quattro	Osseodensification	30.56 ± 1.39	30.67 ± 2.35	29.49 ± 2.37

The data are presented as the mean ± standard deviation; *n* = 10 implants per experimental group. The temperatures correspond to the external surface measurements obtained by infrared thermometry.

**Table 3:** Regional distribution of visually detectable implant thread alterations according to the implant macrogeometry and osteotomy preparation technique.

Osteotomy Preparation Technique	Implant Macrogeometry	Cervical Region, n/N (%)	Body Region, n/N (%)	Apical Region, n/N (%)
Conventional drilling	V2	0/10 (0%)	0/10 (0%)	0/10 (0%)
Conventional drilling	Quattro	0/10 (0%)	0/10 (0%)	10/10 (100%)
Undersized drilling	V2	6/10 (60%)	0/10 (0%)	0/10 (0%)
Undersized drilling	Quattro	6/10 (60%)	0/10 (0%)	8/10 (80%)
Bone expansion	V2	0/10 (0%)	0/10 (0%)	0/10 (0%)
Bone expansion	Quattro	0/10 (0%)	0/10 (0%)	9/10 (90%)
Osseodensification	V2	0/10 (0%)	0/10 (0%)	0/10 (0%)
Osseodensification	Quattro	2/10 (20%)	0/10 (0%)	10/10 (100%)

Data are presented as the number of implants showing visually detectable thread alterations relative to the number examined in each experimental group, with percentages in parentheses; *n* = 10 implants per group.

## Data Availability

The data that support the findings of this study are available from the corresponding author upon reasonable request.

## Abbreviations

AJE	American Journal Experts
CI	Confidence interval
N·cm	Newton-centimeter
rpm	Revolutions per minute
SPSS	Statistical Package for the Social Sciences
η^2^	Eta squared
